# Photosynthesis under far‐red light—evolutionary adaptations and bioengineering of light‐harvesting complexes

**DOI:** 10.1002/1873-3468.70191

**Published:** 2025-10-28

**Authors:** Antonello Amelii, Edoardo Andrea Cutolo, Daniele Montepietra, Claudia Battarra, Roberto Caferri, Stefano Capaldi, Zeno Guardini, Luca Dall'Osto, Roberto Bassi

**Affiliations:** ^1^ Laboratory of Photosynthesis and Bioenergy, Department of Biotechnology University of Verona Italy; ^2^ Stazione Zoologica Anton Dohrn Napoli Naples Italy

**Keywords:** chlorophyll, excitation energy transfer, far‐red light, light‐harvesting, photosynthesis, protein engineering, structural biology

## Abstract

In plants and algae, photosynthesis is driven by the absorption of sunlight energy by networks of pigments housed within light‐harvesting proteins. Special photosynthetic complexes can intercept the low‐energy photons corresponding to the far‐red spectrum of the photosynthetically active radiation. These so‐called red chlorophyll forms are found in multiple lineages of the Viridiplantae clade, are formed upon a change in spatial organization of chromophores within specific subunits of the photosystem I supercomplex, and can be detected by their unique red‐shifted fluorescence emission signatures. Red forms enabled phototrophs to colonize light‐limited ecological niches, especially where far‐red radiation is enriched by leaf shading. The protein environment plays a key role in determining the occurrence of red forms, promoting strong excitonic interactions among chlorophyll *a* molecules and facilitating their excitation by low‐energy photons. In this review, we present a comprehensive account of the evolutionary diversity of long‐wavelength‐driven photosynthesis in eukaryotes, and detail the biophysical and structural determinants of this phenomenon. Finally, we discuss how this knowledge can be applied in biotechnology to engineer crop canopies with broadened light absorption and higher yield potential.

## Graphical abstract

Phototrophs evolved light‐harvesting systems adapted for efficient photon capture in habitats enriched in far‐red radiation. A subset of eukaryotic pigment‐binding proteins can absorb far‐red photons via low‐energy chlorophyll states known as red forms. Their structural and biophysical properties were elucidated through structural and functional studies. This knowledge can be exploited in biotechnology to enhance photosynthesis and yield potential of crops.

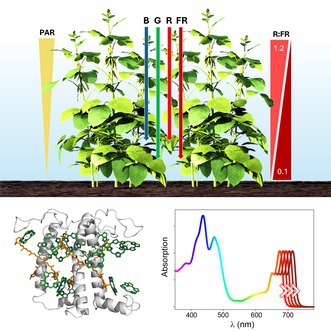

## Abbreviations

**Car**, carotenoid

**CC**, core complex

**Chl**, chlorophyll

**Ct**, charge transfer

**EC**, excitonic coupling

**EET**, excitation energy transfer

**FR**, far‐red light

**HOMO**, highest occupied molecular orbital

**LHCI/II**, light‐harvesting complex I/II

**LUMO**, lowest unoccupied molecular orbital

**PAR**, photosynthetically active radiation

**PSI/II**, photosystem I/II

**R:FR**, red to far‐red ratio

**RC**, reaction center

**RF**, red form

**SC**, supercomplex

## Introduction

**Fig. 1:**
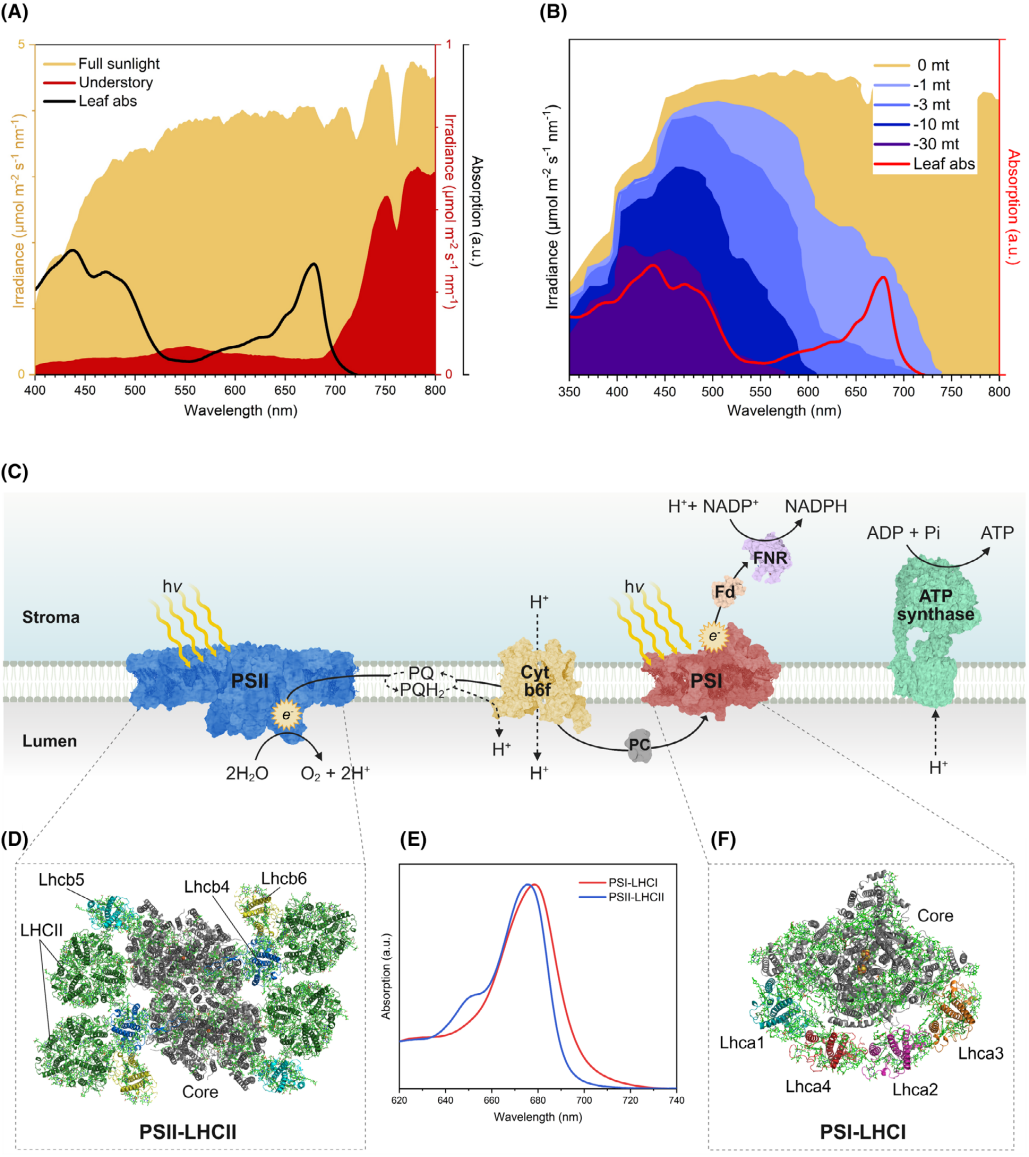
Light spectrum in terrestrial and aquatic habitats, overview of the light reaction of photosynthesis and structure of PSI and PSII supercomplexes. (A) Solar irradiance spectrum at midday recorded at the canopy top (orange) and in the understory (red) (own data). (B) Attenuation of solar irradiance in the water column with depth. This figure was created using data reported in 11. The black (A) and red (B) traces show the absorption spectrum of an *Arabidopsis thaliana* leaf. (C) Schematic overview of the photosynthetic complexes in the thylakoid membrane. Photosystem II (PSII; PDB: 5XNM [11]) captures light energy and catalyzes the oxidation of water, releasing oxygen and transferring electrons to the plastoquinone (PQ) pool. Electrons are transferred to the cytochrome *b₆f* complex (Cyt *b₆f*; PDB: 6RQF [12]), plastocyanin (PC; PDB: 1PNC [13]), and finally to photosystem I (PSI; PDB: 9GBI), which uses additional photon energy to reduce ferredoxin (Fd; PDB: 1A70 [14]). Ferredoxin–NADP^+^ reductase (FNR; PDB: 1FNC [15]) catalyzes the final step in NADPH formation. Electron transfer is coupled to proton translocation across the membrane, generating a proton gradient that powers ATP synthesis via the ATP synthase complex (PDB: 6FKF [16]). The core complexes of PSI and PSII, where charge separation occurs, are associated with peripheral light‐harvesting antennae that enhance photon capture. Whereas the core complexes are highly conserved, the antenna composition varies across photosynthetic lineages. This figure was created with BioRender.com. (D) Structural model of the plant PSII–LHCII supercomplex (PDB: 5XNM), top view. The PSII core complex (Core; gray) is surrounded by peripheral light‐harvesting complexes, including the LHCII trimers and the minor antenna proteins (Lhcb4–6). (E) Absorption spectra of isolated PSII–LHCII (C₂S₂M₂; blue) and PSI–LHCI (red) supercomplexes from *A. thaliana*. PSI–LHCI displays enhanced long‐wavelength absorption compared to the PSII–LHCII (own data, *n* = 3). (F) Structural model of the plant PSI–LHCI supercomplex (PDB: 4XK8), top view. The PSI core complex (Core; gray) is surrounded by the LHCI antenna belt, composed of Lhca1–4 heterodimers (Lhca1 and 4: blue, red; Lhca2 and 3: magenta, orange). Bound chlorophylls and carotenoids are shown in green in panels (D) and (F). The structural models were created using the PyMOL Molecular Graphics System, Version 3.0 Schrödinger, LLC.

Solar radiation drives the photosynthetic reactions that convert light into chemical energy, thereby supporting life on Earth. Evolution optimized the light‐harvesting apparatus of phototrophs to function according to the spectral conditions found on land and in aquatic habitats [1, 2, 3, 4]. In land communities, shading between neighboring plants strongly influences light availability. The upper canopies sequester the red and blue photons and only transmit 1–2% of the incident photosynthetically active radiation (PAR; 400–750 nm) to the forest floor, mainly consisting of the poorly absorbed green and far‐red (FR) wavelengths (Fig. 1A) [5]. Similar steep light gradients are also found within high‐density crop monocultures: following closure, the top canopy is exposed to full irradiance, while the lower foliage experiences constant light limitation. This is also accompanied by a downward decrease of the red‐to‐FR ratio (from 1.2 at the top down to 0.1 at soil level), which limits productivity [6, 7, 8, 9, 10].

In eukaryotic phototrophs, two photosystems [photosystem I (PSI) and PSII] harvest photons through an antenna system consisting of light‐harvesting complex (LHC) proteins that bind chlorophyll (Chl) *a*, Chl *b*, and xanthophylls (Xan). Whereas Chl *a* is present both in the core complexes (CCs) and the peripheral antenna, Chl *b* is only found in the antenna systems [17]. The absorbed energy is funneled to the reaction centers (RCs) of PSI and PSII, where it activates photochemical reactions, initiating a linear flow of electrons along the thylakoid membranes [1]. Electron transport is driven by PSI and PSII, which operate in series, ultimately reducing NADP^+^ to NADPH (Fig. 1C) and sustaining ATP through the building of a trans‐thylakoid proton gradient [18]. PSI and PSII have distinct spectral properties (Fig. 1E): whereas PSII predominantly intercepts blue and red photons, PSI possesses red chl forms or red forms (RFs), which extend its light‐harvesting capacity to cover the longest PAR wavelengths (λ ≥ 700 nm) [19]. Both the PSI core and in its antenna system exhibit a distinct red‐shifted absorption compared to PSII, a property inherited from the cyanobacterial ancestors [20]. The PSI antenna system (LHCI belt) of higher plants consists of two Lhca heterodimers and binds ∼60 Chl molecules (Fig. 1F). These include the RFs, strongly interacting low‐energy Chl pairs absorbing beyond the P700 PSI RC [21, 22, 23, 24]. Although RFs account for a small fraction (4–5%) of photon interception under full sunlight, they contribute up to 40% to photon absorption in shade [25, 26, 27]. Therefore, understanding the structural and biophysical determinants of RFs may provide a blueprint for designing light‐harvesting systems tuned for optimal use of FR light and enhanced photosynthesis in high‐density crop monocultures.

## Far‐red light in plant physiology

The Viridiplantae clade—or green lineage—emerged 1 billion years (Ga) ago. It then witnessed the transition of multicellular phototrophic eukaryotes from marine to land habitats around 500 million years (Ma) ago, and the explosion of angiosperm biodiversity around 110 Ma [28, 29, 30, 31, 32, 33, 34, 35, 36, 37]. Land colonization prompted organisms to adapt to greater environmental variability and a broader light spectrum, leading to competition for photon intercepting. In plants, this was achieved by developing complex canopy architectures and through the expansion of the LHC repertoire to include isoforms optimized for FR light absorption [38, 39, 40, 41, 42, 43, 44, 45, 46, 47, 48]. FR light regulates multiple processes in plant physiology [49, 50]. In the short term, FR light adds to red and blue light in synergistically driving photosynthesis (Emerson enhancement effect) [51, 52, 53, 54, 55, 56, 57] and controls the balancing of PSI *vs*. PSII excitation via reversible post‐translational modifications of LHCs [58]. In the long term, acclimation to FR light causes the reprogramming of pigment metabolism and the adjustment of the chloroplast architecture and of the light‐harvesting apparatus. This includes changes in the PSI/PSII stoichiometry and in the expression of specific LHC isoforms [45, 59, 60, 61, 62, 63, 64]. Over an even longer time frame, FR light acts as a photomorphogenic signal that activates shade‐avoidance growth responses [65, 66, 67, 68]. Therefore, the pleiotropic effects of FR light need to be carefully considered when testing the effect of different light regimes on plant growth responses [69, 70, 71].

### Occurrence of red forms in eukaryotic phototrophs

RFs have been identified within the PSI supercomplex (SC) of most lineages of eukaryotic phototrophs. In contrast, in prokaryotes, RFs primarily result from specific red‐shifted Chl types synthesized during acclimation to FR light, which are incorporated into the CC of both photosystems (for a comprehensive review, see [72]). The evolution of eukaryotic RFs can be traced back to the Ordovician–Silurian period (approx. 489–403 Ma), corresponding to the diversification of vascular plants and the appearance of forest canopies [73, 74, 75, 76, 77, 78, 79]. Eukaryotic RFs arise from pigment‐pigment and pigment–protein interactions within the LHCI belt [80, 81, 82], which, in virtually all higher plants, consists of the protein heterodimers Lhca1–Lhca4 and Lhca2–Lhca3 (Fig. 1F), and displays a characteristic fluorescence emission signature at ∼730 nm at cryogenic temperature (77 K). [22, 83, 84, 85]. Specifically, the *A. thaliana* Lhca1–Lhca4 dimer displays fluorescence emission at 731.5 nm, whereas Lhca2–Lhca3 emits at 728.5 nm [43, 86, 87]. The *in vitro* analysis of recombinant (r)Lhca complexes pinpointed the origin of the red‐shifted fluorescence emission peak to the Lhca3 (725 nm) and Lhca4 (733 nm) subunits, whereas rLhca1 and rLhca2 both emit at 702 nm [88, 89, 90, 91, 92, 93]. Furthermore, the mutational analysis of rLhca subunits revealed that the absorption tail causing the red‐shifted fluorescence emission in Lhca3 and Lhca4 originates from strong excitonic interaction of the Chl pair *a*603–*a*609, where the asparagine (Asn) residue coordinating Chl *a*603 emerged as the key structural determinant (described in more detail in Section ‘The protein ligand’) [43, 94, 95]. Both emission and absorption of LHCI subunits display a more pronounced red‐shift when they are assembled in dimeric structures and when they are associated with the PSI CC [96]: the fluorescence emission λ_max_ of Lhca3 shifts from 725 nm to 728.5 nm when part of the Lhca2–Lhca3 dimer and to 735 nm in the fully assembled PSI–LHCI complex [86, 87, 97, 98, 99, 100]. These effects derive from the combination of multiple factors, including pigment–pigment and pigment–protein interactions, which stabilize these low‐lying energy states (discussed in Section ‘Biophysical determinants’) [101].

**Fig. 2:**
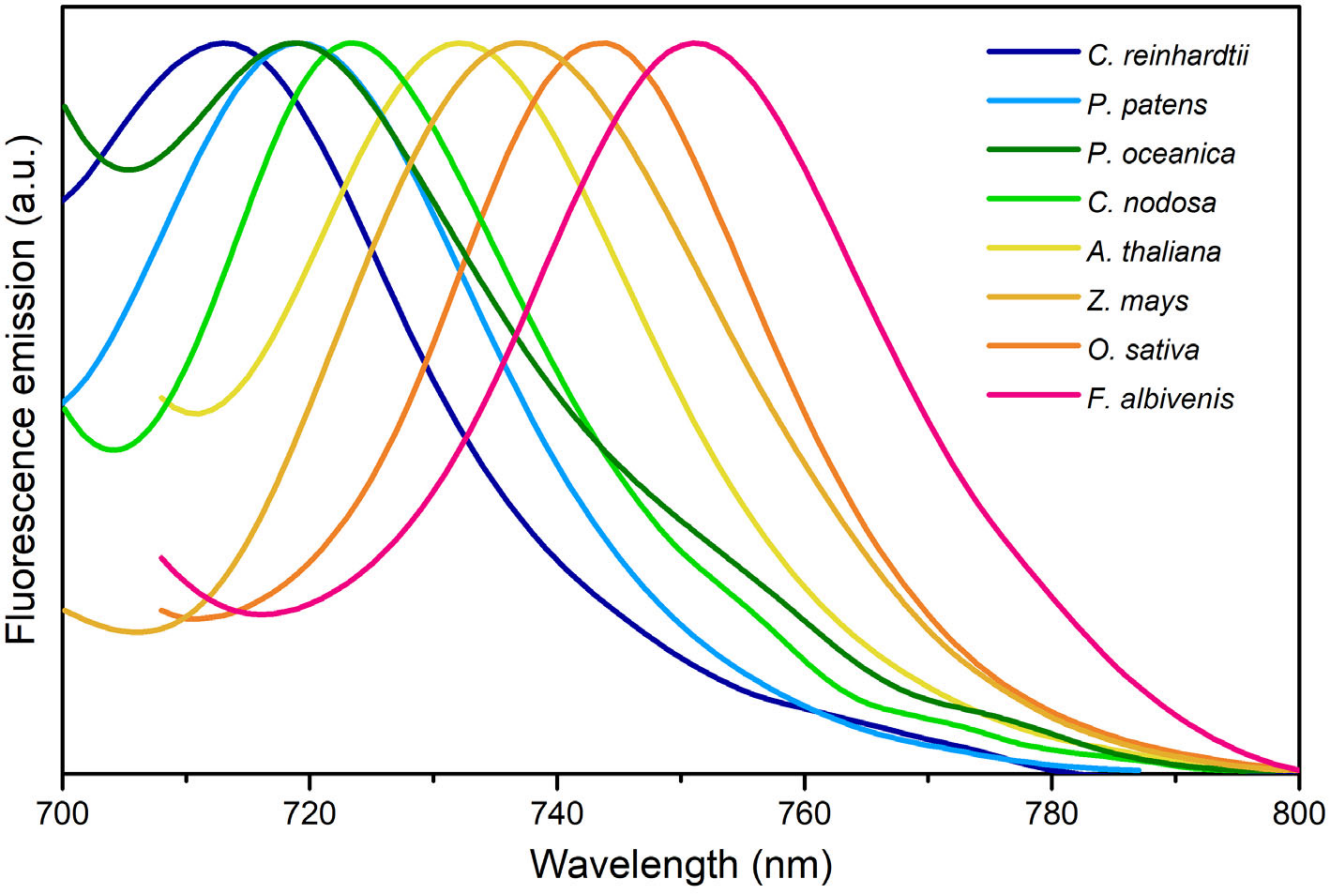
Progressive extension towards the red spectrum of light‐harvesting systems in the evolution of eukaryotic phototrophs. Fluorescence emission spectra were recorded at cryogenic temperature on leaf extracts and cell suspensions of representative species from phylogenetic groups. This figure was created using data from [106].

Plant evolution was accompanied by the constant expansion of PSI absorption toward longer wavelengths, and the progressive relocation of RFs from the CC to peripheral LHCI sites [25, 26, 37, 84, 98, 102, 103, 104, 105] (Fig. 2). Red‐shifted emission spectra between 730 and 740 nm were recorded in land plants such as *Nicotiana tabacum* (Solanaceae), *Zea mays* (Poaceae), *Pisum sativum* (Fabaceae), *Spinacia oleracea* (Amaranthaceae), *Spathiphyllum wallisii* (Araceae), *Calathea roseopicta* (Marantaceae), and *Hordeum vulgare* (Poaceae) [98, 99]. Greater shifts were observed in *Oryza sativa* (743 nm; Poaceae) and *Panax notoginseng* (747 nm; Araliaceae), and even more in the shade‐tolerant species *Fittonia albivenis* (Acanthaceae), with λ_max_ values beyond 748 nm [100]. Intriguingly, the seagrasses *Posidonia oceanica* (Posidoniaceae) and *Cymodocea nodosa* (Cymodoceaceae) exhibit blue‐shifted emission peaks at around 720 nm and 723 nm, respectively [106]. The loss of RFs in these species probably occurred because of the relaxed selective pressure following their return to the FR‐deprived marine environment approx. 60 Ma (Fig. 1B) [107, 108, 109, 110]. Furthermore, the PSI–LHCI emission λ_max_ values of most extant mosses (bryophytes) range between 717 and 735 nm [37, 82, 111]. Mosses are evolutionary intermediates between green algae and land plants and display early adaptations to land shade environments, including unique PSI features [29]. For instance, in *Physcomitrium patens*, the Lhca4 subunit is replaced by the paralog isoform Lhca2b. In Lhca2b, Chl *a*603 is coordinated by a histidine (His) residue, a modification that likely accounts for a ∼10 nm shorter emission wavelength compared to the PSI–LHCI complexes of higher plants [37]. Instead, in the Lhca3 subunit, Chl *a*603 is coordinated by Asn like in angiosperms, suggesting that this feature was established during the adaptation to FR‐light‐enriched environments [82]. Another specific feature of *P. patens* is the Lhcb9 subunit found in the PSI–LHCI–LHCII megacomplexes, in which both Chls *a*603 and *a*612 are coordinated by Asn residues and display an emission peaking between 683 and 687 nm [112, 113, 114, 115, 116].

Blue‐shifted PSI–LHCI fluorescence emission peaks are observed further up in the phylogenetic tree of phototrophic eukaryotes. For instance, the freshwater unicellular chlorophyte *Chlamydomonas reinhardtii* displays emission λ_max_ around 720 nm [104, 117]. In this species, RFs are found in the outer LHCI belt, where the Lhca2, Lhca4, and Lhca9 subunits occupy a more distal position from the CC compared to the Lhca3 and Lhca4 plant isoforms. In these subunits, Chl *a*603 is coordinated via conserved Asn residues [81, 104]; however, they display a blue‐shifted emission peaking between 690 and 717 nm, respectively [117]. Similarly, in the marine chlorophyte *Ostreococcus tauri*, Asn residues coordinate Chl *a*603 in Lhca5 and Lhca6, but their emission λ_max_ peaks at ~700 nm [118], suggesting that other protein features influence their spectral responses [41, 119].

Recently, additional types of RFs have been described in the eustigmatophyte *Trachydiscus minutus* [120]. In this species, a cluster comprising Chl *a* and two Xan molecules and exhibiting strong excitonic interaction is found in a specific class of LHCs known as violaxanthin–Chl *a* proteins [121, 122]. Furthermore, in the trebouxiophyte *Prasiola crispa*, a long‐wavelength Chl *a* accumulates in its PSII–LHCII [123]. These features require the FR‐light‐induced expression of the *Pc‐frLHC* gene, a divergent LHC isoform [124]. This type of acclimation response is observed in other phytoplankton taxa, as recently reported for the flagellate *Euglena gracilis*. In this case, pentameric complexes consisting of the taxon‐specific FR‐absorbing Lhce isoform associate with both PSI and PSII under low light following FR light acclimation [125, 126, 127]. Finally, in some diatoms and other eustigmatophytes, the occurrence of RFs appears to depend on additional factors other than pigment geometries, such as the Chl:carotenoid ratio of LHCs, highlighting the complex basis of low‐energy photon absorption in photosynthesis [120, 128, 129].

## Principles of low‐energy absorption

### Biophysical determinants

**Fig. 3:**
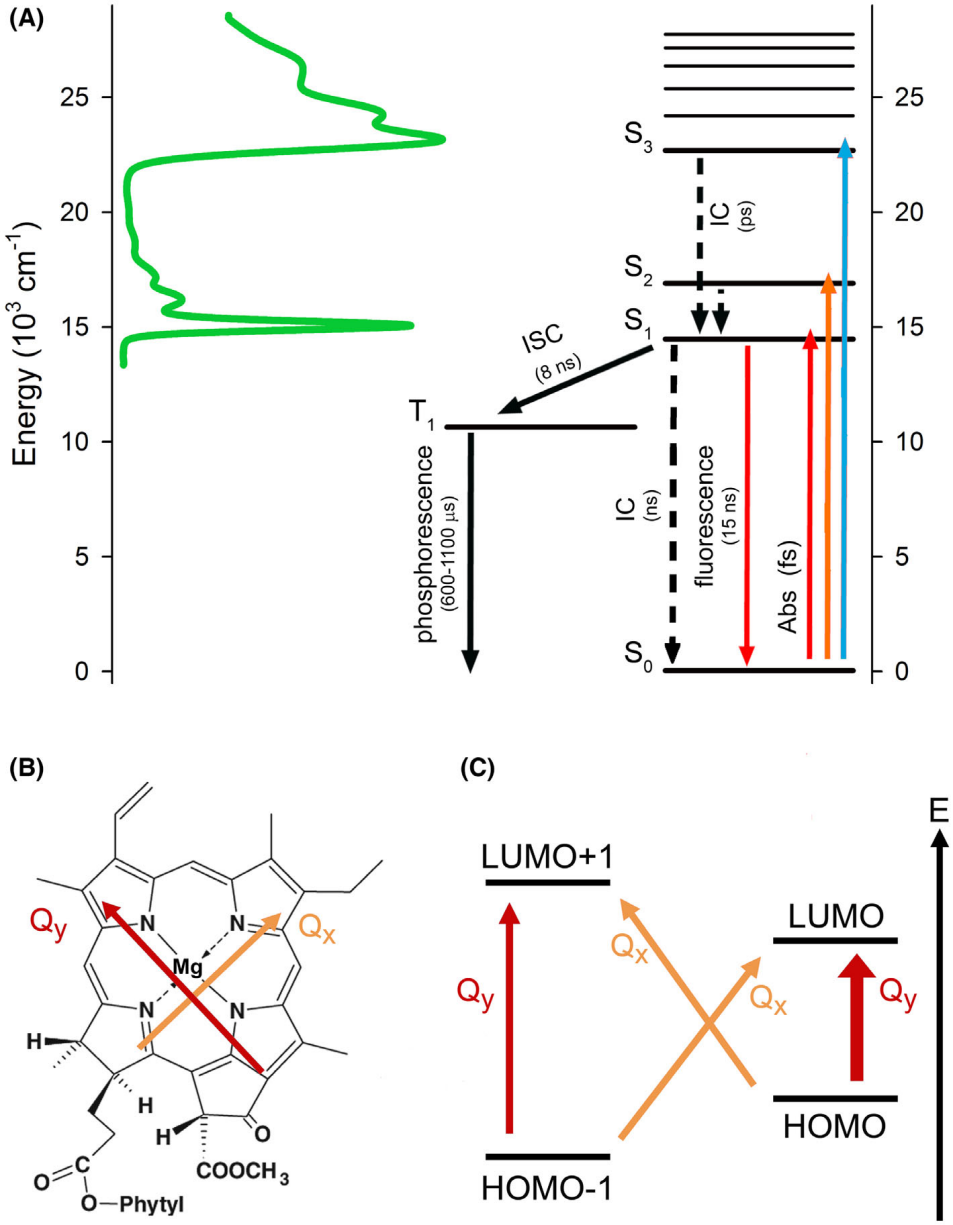
Transition energy diagram of chlorophylls. (A) Chl *a* transition energy diagram. Excitation and relaxation processes are indicated by arrows and their corresponding time constants. The range of energies for pigment transitions is related to the visible absorption spectrum of the pigment. Abs, absorption; IC, internal conversion; ISC, intersystem crossing. (B) Chemical structure of Chl *a*. The red and orange arrows indicate the orientations of the Q_x_ and Q_y_ transition dipole moments, respectively. (C) Contributions of the Highest Occupied Molecular Orbital (HOMO), the second HOMO (HOMO–1), Lowest Unoccupied Molecular Orbital (LUMO) and the second LUMO (LOMO+1) to the Q_y_ and Q_x_ absorption bands. The Q_y_ transition is represented as a linear combination of transitions from HOMO to LUMO and from HOMO–1 to LUMO+1, whereas the Q_x_ transition is expressed as a linear combination of transitions from HOMO–1 to LUMO and from HOMO to LUMO+1. The thick red arrow indicates the main Q_y_ transition. This figure has been adapted from [134, 135].

Chls are tetrapyrrole macrocycles that incorporate an Mg^2+^ ion, a phytol chain, and a characteristic fifth ring, together featuring an extended system of conjugated bonds that is responsible for two main absorption bands in the blue (Soret band) and red (Q band) regions of the visible spectrum. These bands comprise independent electronic transitions named Q_y_ (or S_1_), Q_x_ (or S_2_), and the Soret band (S_3_), which is a superposition of the B_x_ and B_y_ bands, with the x and y annotations indicating the polarization directions within the macrocycle plane (Fig. 3A) [130]. In Chl *a*, the lowest (Q_y_) and the second lowest energy (Q_x_) absorption bands possess orthogonal transition dipole moments. These transitions are qualitatively described by the four‐orbital model, which involves the two highest occupied molecular orbitals (HOMOs) and the two lowest unoccupied molecular orbitals (LUMOs) [131, 132, 133]. The Q_y_ transition can be expressed as a linear combination of the transition from HOMO to LUMO and from HOMO−1 to LUMO+1. In contrast, the Q_x_ transition is expressed as a linear combination of transitions from HOMO−1 to LUMO and from HOMO to LUMO+1 (Fig. 3C). The B_x_ and B_y_ transitions can also be expressed by linear combinations of the two transition dipole moments along the *x*‐ and *y*‐axes, respectively (Fig. 3B) [134].

The absorption spectra of Chl‐binding proteins exhibit significant heterogeneous broadening in the Q_y_ region (630–685 nm range) due to the presence of distinct spectral forms [136], which are attributed to the localization of Chls in different protein sites. The interactions between Chls *a* and their protein environment modulate the pigment absorption properties compared to organic solutions. These interactions cause spectral broadening and a shift of their absorption toward longer wavelengths (bathochromic shift) due to changes in electronic energy levels. Specifically, the Q_y_ band shifts from 664 nm in organic solvents to the 665–685 nm range observed in most Chl *a* molecules when bound to photosystems [137]. The arrangement of neighboring pigments can further shift Chl absorption into the red spectrum. For instance, some molecular interactions that produce RFs result in energy states lower than the primary photochemical trap of the PSI RC. This spectral red‐shift arises from excitonic coupling, in which the excited states are delocalized over two or more pigments [92, 138]. This coupling is highly sensitive to the environment, so slight structural differences in the host protein can alter the geometry of pigment organization, bringing the Chls closer together. This enhances the probability of inter‐pigment electron transfer processes, making charge transfer (CT) states more significant. Since the CT states are generally close in energy to the singlet‐excited states of a Chl pair, they will tend to mix into the excited state. These events can significantly change the spectroscopic properties of pigment–protein complexes, leading to large homogeneous broadening, Stokes shifts, and strong electron–phonon coupling [92, 139, 140].

#### Excitonic coupling

Chls spaced closely together can form molecular excitons, which are excited electronic states involving more than one pigment molecule. Exciton energies result from the coupling between the excited states of individual pigments and are affected by their local environment—including nearby residues, protein electrostatics, and the medium. Although excitons are delocalized states, their properties depend on the energies of localized excitations and the interactions between them [141].

The electronic properties of pigment aggregates are commonly described by the Frenkel exciton model, in which the excitonic coupling (EC) can be decomposed into a long‐range Coulomb contribution and a short‐range term related to the orbital overlap between interacting chromophores (CT contributions) [142, 143, 144]. The exciton model typically neglects all CT contributions to the aggregate excited states. This simplification is justified by the fact that most interpigment distances are sufficiently small to enable significant EC, yet sufficiently large to prevent CT effects. By omitting CT contributions, a classical interpretation of the system can be provided, as the electrostatic interaction between two charge densities represents the excitations of the coupled chromophores [145].

EC becomes relevant when the interaction energy *E*_ab_ is proportional to or exceeds the energy difference between the individual pigments (*i.e*., *E*_*ab*_ > |*E*_*a*_ − *E*_*b*_|). Conversely, when excitonic mixing is moderate or minimal (i.e., *E*_*ab*_ < |*E*_*a*_ − *E*_*b*_|), excitations are largely localized over one or more Chls that make up the aggregate, allowing the Förster equation to remain applicable. This is evident from the observation that the strongest EC between two Chls is ∼120 cm^−1^, thus significantly smaller than the energy separation between the Q_y_ levels of these molecules (∼450 cm^−1^). ECs are quite stable and, therefore, less sensitive to changes of the internal geometry and orbital overlap of pigments. ECs are more strongly influenced by the relative position and orientation of pigments, since they are determined by the Coulomb coupling between transition dipole moments. By contrast, site energies can vary significantly with minor changes in molecular geometry [140].

#### Charge transfer

When pigments lie in proximity, the likelihood of electron transfer between them increases, making CT states significant. These CT states are polar in nature, exhibiting an excited‐state dipole moment between the ground and excited states [92, 146]. The dipole moment associated with CT states facilitates coupling between the optical transition and the (polar) phonon (i.e., quantum of vibrational mechanical energy) of the environment, thereby enhancing exciton–phonon coupling. Stark spectroscopy experiments revealed that the excitonic states of Chl *a* in LHCII probably behave in a similar way to those of unbound monomeric Chl *a* [147]. This indicates that there is little or no delocalization of the excitation, which would be expected in the case of strong EC [147].

The absorption spectrum of a pigment within a protein coordination site typically displays a narrow zero‐phonon line, representing the pure electronic transition, along with a broad, blue‐shifted phonon band associated with low‐frequency vibrations of the protein matrix, coupled to the electronic transition [148]. Several factors influence the width of these absorption bands. For instance, the coupling of electronic transitions to vibrations of the pigments and the protein environment (phonons) leads to homogeneous broadening, which is temperature‐dependent [149].

By contrast, inhomogeneous broadening arises from structural fluctuations within the protein matrix, leading to a Gaussian distribution of optical transition frequencies. This statistical distribution remains unaffected by temperature but is influenced by slow fluctuations that alter the pigment‐binding sites [92, 150]. The magnitude of CT couplings, which is directly related to the degree of molecular orbital overlap [140], is crucial for the occurrence of low‐energy absorption and fluorescence emission signatures. By contrast, the energies of CT states, which are significantly higher than the exciton energies, primarily influence the precise positions of the red‐shifted bands [140].

EC alone, however, does not fully account for the spectra of red‐most LHC proteins, such as Lhca4, or for the broad bandwidth associated with RFs. For instance, the red‐most transition of LHCII, which has a similar pigment organization but an interaction energy that is half that of Lhca4 (120 cm^−1^ *vs*. 260 cm^−1^) is centered around 681 nm [92, 149, 151]. In Lhca4, both Chl *a*610–*a*611–*a*612 and *a*603–*a*609 pigment clusters are red‐shifted. However, the lowest exciton level of the *a*603–*a*609 cluster lies further in the red spectrum due to mixing with the charge transfer state [95, 138, 140, 152, 153, 154, 155]. As suggested by recent quantum mechanics/molecular mechanic models focusing on the Lhca4 monomer, a larger component of CT displays *a*603 ➔ *a*609 directionality [106].

#### Excitation energy transfer

Within the LHC protein matrix, pigments create local funnels that convey the absorbed energy to the RCs through preferential routes [1, 42, 156]. Pigments exhibit different site energies, which are tuned by the interactions with neighboring chromophores and, in turn, govern the excitation energy transfer (EET) dynamics. RFs constitute energy valleys lying deeper than RCs that influence EET in PSI–LHCI, decreasing the overall trapping rate [157, 158]. Significantly, the slow energetically uphill EET pathway from RFs in LHCI increases the trapping time by a factor of three: from ~22 ps to ~65 ps [86, 87, 159, 160].

An important distinction exists between PSI antenna subunits with few but highly intense red clusters (Lhca3 and Lhca4) and those featuring multiple red clusters with higher energy levels (Lhca1 and Lhca2), in terms of EET and trapping times [158, 161, 162]. For instance, the red Lhca3 and Lhca4 isoforms are less efficient in transferring energy to the RC compared with the blue isoforms Lhca1 and Lhca2 (40 *vs*. 10 ps), with Lhca3 contributing approximately 40% to the increased trapping time in PSI–LHCI, whereas Lhca4 accounts for 60% [87].

In the antenna subunits that contain more isoenergetic Chl clusters (Lhca1 and Lhca2), excitons can populate several of the lowest energy sites after equilibration. For instance, the lowest exciton level of the Chl *a*602–*a*603–*a*609 cluster of Lhca1 is also red‐shifted, albeit to a lesser extent than the same cluster of Lhca4. Furthermore, Lhca1 contains additional RFs as part of the Chl *a*610–*a*611–*a*612 cluster, as well as on its CC‐exposed side, consisting of Chl *a*606 and the *a*613–*a*616 pair. Their coupling has been suggested to facilitate the depopulation of Lhca4, providing an alternative EET route to the CC [89, 163].

**Fig. 4:**
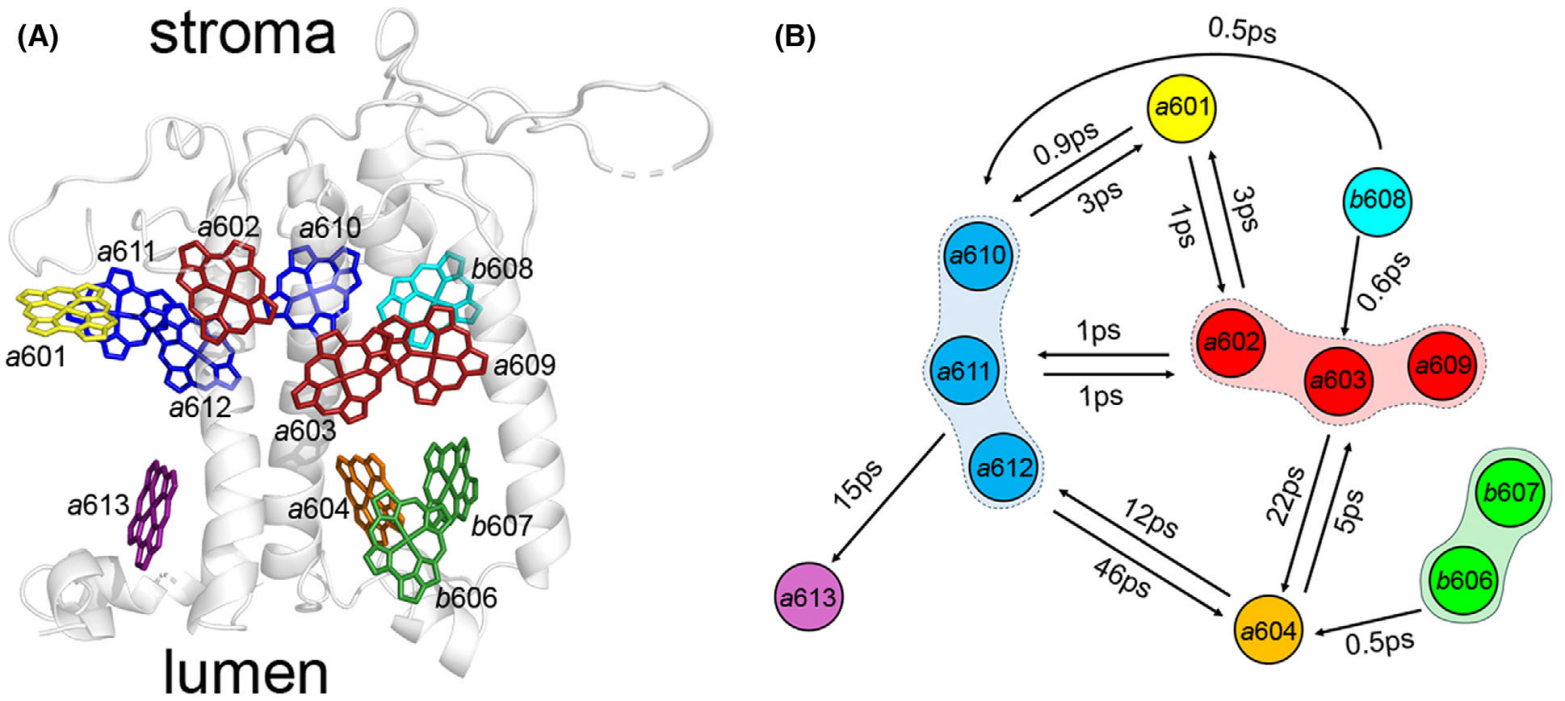
Pigment topology and energy transfer pathways in the Lhcb4 subunit of photosystem II. (A) Illustration of the Chls embedded within the protein scaffold of the PSII monomeric antenna complex Lhcb4 (CP29). The structure is based on [169] and shows the nomenclature of Chls housed inside Lhcb4 and the complex orientation with respect to the thylakoid membrane. (B) Excitation energy transfer between the pigment domains of Lhcb4 based on [169].

It has also been proposed that low‐energy clusters may exist within the PSII antenna system (Lhcb). For instance, within trimeric LHCII complexes, an efficient EET is facilitated by the presence of the red Chl cluster *a*610–*a*611–*a*612. Notably, Chl *a*610 has been identified as the red‐most site, where energy is predominantly populated, thereby promoting a functional EET route towards other PSII subunits and the CC [42, 164, 165, 166, 167, 168]. Another preferential EET route to the PSII RC involves two low‐energy sites (*a*610–*a*611–*a*612/*a*602–*a*603–*a*609) located in the monomeric antenna Lhcb4, which create a structural and energetic bridge at the interface between the outer LHCII system and the CC (Figs 1D, 4) [169]. Instead, two Chl pools with energy levels lower than PSII RC (P680) are housed in the inner antenna subunits CP43 and CP47 of the PSII CC, which are characterized by fluorescence emission at 685 nm and 695 nm, respectively [170, 171].

### Structural determinants

#### The protein ligand

**Fig. 5:**
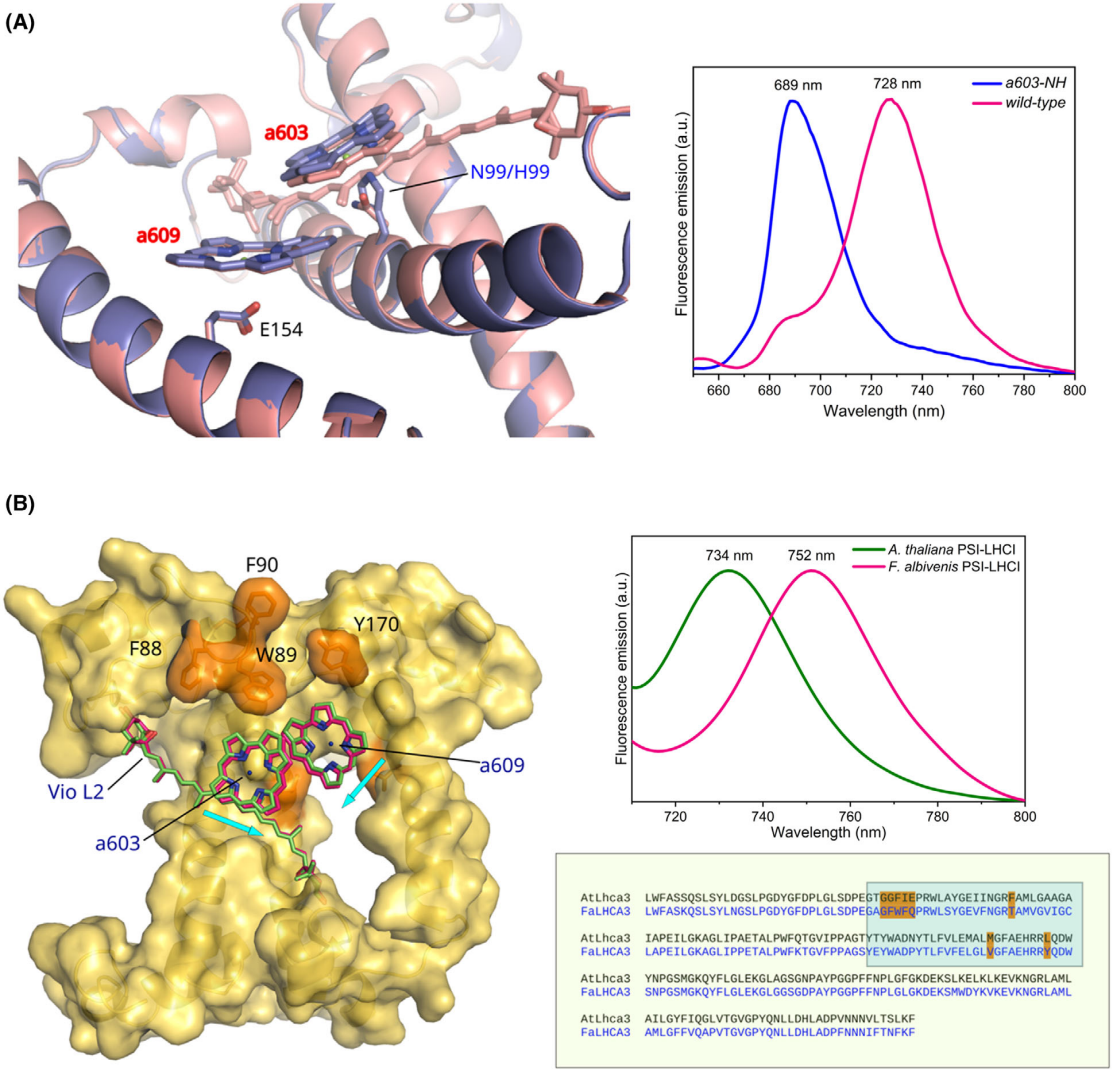
Structural determinants of low‐energy absorption in the Lhca3 and Lhca4 subunits of the PSI–LHCI supercomplex of higher plants. (A) Position of Chls *a*603 and *a*609 in the wild type (salmon; PDB 9GBI) and in the Asn (N)99‐His (H) mutant (blue; PDB 9GC2) Lhca4 subunit of the *A. thaliana* PSI–LHCI supercomplex. The effect of the mutation on fluorescence emission at 77 K of isolated Lhca heterodimers is shown in the right box and is based on [106]. (B) The presence of a group of bulky hydrophobic residues in the vicinity of the Chl pair *a*603–*a*609 (red) in Lhca3 of *Fittonia albivenis* (PDB 8WGH [100]) causes a subtle displacement of the chlorine rings compared to *A. thaliana* (green), causing a red‐shifted fluorescence emission (right). The lower box displays the sequence alignment between the Lhca3 isoforms of *A. thaliana* and *F. albivenis*, highlighting the short hydrophobic amino acid stretch and three residues that potentially contribute to the strongly red‐shifted fluorescence emission (highlighted in orange inside the blue box). Cyan arrows indicate the displacement of chromophores due to differences in the local protein environment. This figure was created using The PyMOL software/Molecular Graphics System, Version 3.0 Schrödinger, LLC. The multiple sequence alignment was performed using the Clustal Omega (EMBL‐EBI) [175] using the Lhca3 amino acid sequences retrieved from PDB 8WGH (*F. albivenis*) [100] and PDB 9GBI (*A. thaliana*).

The identity of the amino acids that coordinate Chl *a*603 and *a*612 is a key factor in determining the occurrence of RFs in the Lhca subunits of *A. thaliana* (Fig. 5A) [43, 95, 140, 172]. In the monomeric PSII antenna Lhcb4, two His residues are responsible for this coordination, and the absorption λ_max_ is 680 nm [169, 173, 174]. Conversely, in Lhca1 and Lhca2, where Asn residues coordinate Chl *a*612 and His residues coordinate Chl *a*603, λ_max_ is shifted to 702 nm. Lhca3 and Lhca4, however, feature two Asn residues coordinating Chl *a*603 and *a*612, resulting in a λ_max_ greater than 720 nm [95]. In *P. patens*, which has an Asn residue in Lhca3 and a His residue in Lhca4 as Chl *a*603‐binding residues, a less red‐shifted emission is observed compared to higher plants [43]. As previously mentioned, some angiosperms display different λ_max_ despite sharing the same ligands of the Chl pairs [98], probably as a consequence of the different orientations of the transmembrane helices and local protein environments, which affect the interactions between the bound pigments. Therefore, these interactions potentially make a more significant contribution to RF occurrence than the identity of the coordinating ligand [43]. Although the coordination of Chl *a*603 by Asn is not sufficient to confer significant FR absorption, it plays a critical role in maintaining the correct geometry between Chl *a*603 and *a*609, which is essential for the strong interaction in this cluster [43, 84, 140]. Indeed, when Asn is replaced by His or Gln, the corresponding RF is lost [43, 95].

#### The protein environment

The protein scaffold that houses Chls influences directly and indirectly the width of the absorption bands of pigments. For instance, the environment may include charged residues that promote hydrogen (H)‐bonds between the amino acid side chains and pigment functional groups [43, 176, 177]. Moreover, the protein backbone stabilizes pigments, modulates their interactions, and provides vibrations at appropriate frequencies, bridging energy gaps between excitonic states that facilitate EET [1, 84, 150, 178]. Notably, the protein environment of the red‐shifted Chl cluster *a*603–*a*609 is different. Whereas Chl *a*609 is immersed in a charged environment via the coordination by glutamate (Glu) and an adjacent arginine (Arg), Chl *a*603 is mainly surrounded by apolar residues. It was suggested that the presence of bulky hydrophobic amino acids close to the Chl *a*603–*a*609 cluster of Lhca3 (phenylalanine–tryptophan–phenylalanine in place of glycine–phenylalanine–isoleucine) may contribute to the large red‐shift in *F. albivenis* and other Acanthaceae (*e.g*., *Strobilanthes cusia* and *Andrographis paniculata*) (Fig. 5B) [100]. The steric hindrance caused by the side chains of these hydrophobic residues appears to reduce the distance between the chlorine rings of Chls *a*603 and *a*609, promoting their stronger interaction [100]. In addition, the relative orientation between Chl *a*603–*a*609, combined with changes in the chemical and physical properties of the environment, might further reinforce the red‐shifted fluorescence emission recorded in these species (Fig. 5A).

It was suggested that additional chromophores may contribute to low‐energy absorption in PSI–LHCI subunits. For instance, the extra Chl *a*615 (also known as *a*617) was assigned to the Lhca4 subunit of land plants based on structural homology with the LHCII monomer [179]. Its presence has been experimentally confirmed through structural analysis of the PSI–LHCI SC of *P. sativum* (PDB 7DKZ) [22], *Z. mays* (PDB 5ZJI) [180] and *F. albivenis* (PDB 8WGH) [100]. However, recent mutational studies conducted *in vivo* have excluded its direct involvement in the formation of RFs [106].

Some reports have suggested that Chl *b* may also contribute to the stabilization of RFs in Lhca4, although the exact identity of the pigments involved is still unknown [90, 93, 181].

As previously mentioned, some eukaryotic microalgae display different types of PSII outer antennae that enable FR light absorption. For instance, the freshwater species *P. crispa* (Trebouxiophyceae) possesses a ring‐shaped endecameric LHCII system consisting of four‐helix Pc‐frLHC monomers in addition to the canonical trimeric arrangement of three‐helix LHCs [182, 183]. These circular complexes display a red‐shifted absorption around 710 nm originating from the strongly interacting Chl *a*603–*a*609 pair housed inside the Pc‐frLHC subunits, which is responsible for a large excitation‐induced permanent dipole moment between the two Chls with a pronounced CT character. In addition, Chl *a*609 appears to be excitonically coupled with the neighboring Chl *a*708, possibly forming a Chl trimer, which provides the structural basis for FR light absorption. Finally, more subtle alterations of the protein environment appear to enable FR light absorption by the only Chl *a*‐based LHC system in some eustigmatophytes [184, 185].

## Bioengineering low‐energy absorption in plants

**Fig. 6:**
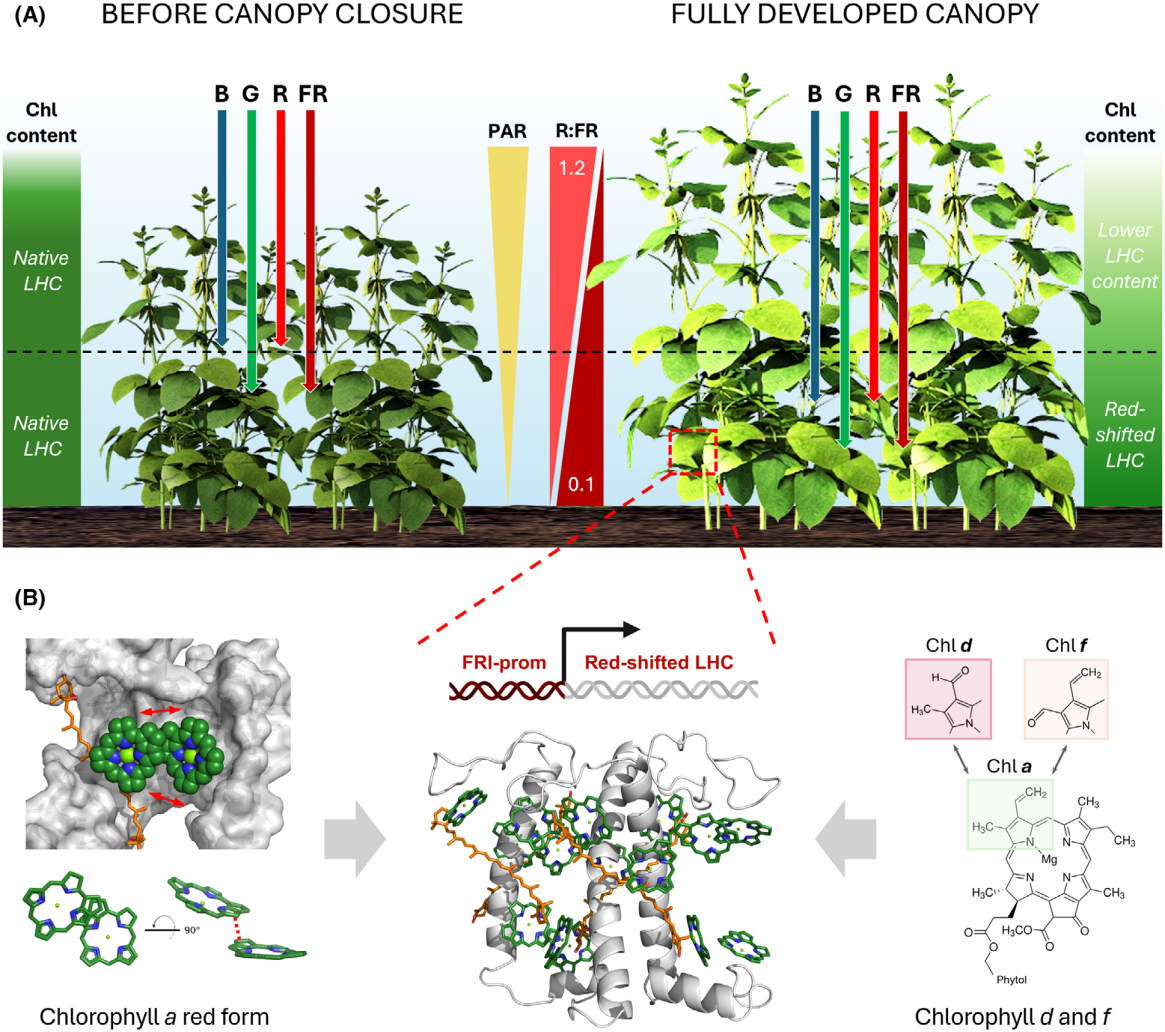
Proposed ‘smart canopy’, engineered to optimize the efficiency of FR light use in dense crop monocultures. Panel (A) illustrates two stages of canopy growth. Left: Before canopy closure, the light‐harvesting apparatus consists of a native complement of LHC proteins, and leaf chlorophyll (Chl) content is homogeneous across leaf layers. In this configuration, the top leaves act as a filter for incoming photosynthetically active radiation (PAR), limiting the penetration of blue (B) and red (R) photons. Therefore, the lower leaf layers receive predominantly green (G) and far‐red (FR) wavelengths, as reflected by a markedly reduced R:FR ratio. Right: In a fully developed canopy, a gradual reduction in Chl content from the top to the bottom foliage should promote a more uniform light distribution within the canopy. The predominantly FR light environment in the lower layers could act as a trigger to induce the expression of red‐shifted LHCs, to enable efficient utilization of the available spectrum. Panel (B): A feasible strategy to enhance low‐energy absorption involves using FR‐inducible (FRI) promoters to drive expression in the lower leaf layers of red‐shifted LHCs incorporating chlorophyll *d* and *f* or carrying structural modifications such as novel pigment–protein and pigment–pigment interactions. Centre: conserved structure of an LHC protein (Lhca4; PDB: 9GBI) with bound chlorophylls (green) and carotenoids (orange), showing their organization within the scaffold (gray). Left: Close‐up view of inter‐pigment interactions within LHCs, highlighting Chl *a* molecules, their coupling, and the geometry influenced by the binding site and local protein environment. Right: Chemical structures of Chl *a*, *d*, and *f*. The structural models in this figure was created using The PyMOL Molecular Graphics System, Version 3.0 Schrödinger, LLC.

Rational genetic engineering has the potential to significantly accelerate the development of elite crop cultivars by enhancing their resource‐use efficiency [186]. Among various traits, photosynthesis is an emerging target for crop improvement, with multiple strategies proposed to increase the efficiency of sunlight‐to‐biomass conversion (for detailed reviews see [187, 188, 189, 190, 191]). Bioengineering the absorption of low‐energy photons in plants can expand the light‐harvesting capacity of leaves, thereby boosting photosynthesis under light conditions enriched with longer wavelengths, for example beneath dense canopies [192, 193]. Ideally, a “smartly designed” crop canopy should facilitate the uniform distribution of wavelengths throughout its vertical profile [194] (Fig. 6). This could be achieved, for example, by regulating the leaf angle, or by lowering leaf Chl content to decrease the canopy optical density [195, 196, 197]. Alternatively, the light‐harvesting apparatus of shaded foliage could be tuned to efficiently utilize the available light spectrum, particularly by maximizing the interception of photons in the red‐most PAR region [196].

Independent studies have suggested that, by fully harnessing the absorption of the 700–750 nm range, energy conversion in crops could be enhanced between 19 and 26% [192, 193, 198], representing a significant improvement compared to the estimated 4–6% efficiency observed in most crop species [199]. This bioengineering concept is eliciting great interest and could be achieved through a combination of approaches (Fig. 6).

One strategy involves incorporating red‐shifted prokaryotic Chl types (Chl *d* and *f*) into eukaryotic LHC complexes, to enable excitation by photons with lower energy than those typically absorbed by Chl *a* and *b* (for a dedicated review, see [72]). The feasibility of this approach has been demonstrated *in vitro*, where Chl *d* and *f* could be incorporated into the plant Lhca4 protein, causing a pronounced red‐shift without compromising its functions and structural integrity [200]. Moreover, Chl *d* has been successfully integrated into the plant LHCII complex *in vitro*, producing an ∼25 nm absorption red‐shift while maintaining the native protein architecture, EET and energy quenching [201, 202]. However, the implementation of heterologous Chl biosynthesis in plants remains unreported and faces at least three major challenges: (i) the factor(s) required for Chl *d* synthesis are yet to be identified [203, 204], (ii) the *in vivo* incorporation of non‐native Chl types into eukaryotic LHCs may be suboptimal [205] and (iii) inserting low‐energy pigments into Chl *a*‐dominated eukaryotic LHCs could create energy traps, thereby slowing EET and energy trapping, especially in the PSII RC [206].

A second strategy, discussed in the next paragraph, aims at broadening the absorption capacity of light‐harvesting systems by modifying the native pigment–pigment and pigment–protein interactions through engineering of the protein scaffold to create novel spectral properties of LHCs.

### Spectral tuning of LHCs through novel pigment–protein interactions

The spectral properties of LHCs can be modified to enhance FR light absorption via substitutions of key chlorophyll‐binding residues and/or altering the local protein microenvironment. Such changes influence the spatial arrangement (distance and orientation) of pigment pairs, thereby affecting pigment–pigment and pigment–protein interactions. This, in turn, shifts the balance between EC and CT states. Since excitonic interactions are highly sensitive to the local environment, even subtle structural modifications of the protein scaffold can reshape pigment geometry, leading to notable changes in the protein spectral responses [207].

LHC proteins exhibit a highly conserved three‐dimensional architecture characterized by three transmembrane helices interconnected by amphiphilic loops [208]. In plant LHCs, at least six conserved residues are involved in Chl coordination, along with other key residues that shape the local protein microenvironment. Several of these residues are thus promising targets for mutagenesis [41].

The first proof‐of‐concept study reporting the *in vivo* engineering of these interactions was performed in the photosynthetic purple bacterium *Rhodobacter sphaeroides*. This work established a direct correlation between the absorbance properties of bacteriochlorophyll *a* and two coordinating tyrosine residues (Tyr44 and Tyr45) in the α subunit of the peripheral light‐harvesting complex LH2 [176, 209]. The single and double substitution of Tyr44 with Phe and Tyr45 with Leu caused blue‐shifts of 11 and 24 nm, respectively. The same rationale was employed *in vitro* with the plant subunits rLhca3 and rLhca4, leading to the identification of the Asn ligand of Chl *a*603 as the structural determinant responsible for the red‐shifted emission [95]. This approach was subsequently tested in *A. thaliana* by replacing the Asn coordinating Chl *a*603 in the Lhca4, producing a 2–3 nm blue‐shift of the emission peak at 77 K of the isolated PSI–LHCI SC [172]. Instead, the double substitution of Asn with His in both Lhca3 and Lhca4 resulted in a greater blue‐shift, around 13 nm, in the isolated PSI–LHCI SC and a 39 nm shift in the isolated LHCI heterodimers [106]. Intriguingly, replacing a Glu residue located in the transmembrane helix with Gln slightly enhanced the native red‐shift [43, 172].

A similar *in vivo* strategy was used with the PSII monomeric antenna protein Lhcb4 (CP29) in *A. thaliana*, in which the His residue coordinating Chl *a*603 was substituted with Asn (His111Asn) [173, 174]. Despite the marked structural similarity between Lhcb4, Lhca3, and Lhca4, this mutation resulted in an ~14 nm red‐shift in the 77 K fluorescence difference spectra compared to the wild‐type, which is substantially lower than the ~39 and 44 nm shifts observed in Lhca3 and Lhca4, respectively [95, 173, 174]. Furthermore, the calculated difference in absorption spectra (Lhcb4 wild‐type minus His/Asn mutant) revealed a conserved signal in the Q_x_ region, with a negative peak at 676 nm and a positive peak at 686 nm [173]. This 10 nm shift was attributed to enhanced EC between the Chl pair *a*603–*a*609. Moreover, the mutant Lhcb4 isoform displayed a shorter fluorescence lifetime and a reduced formation of carotenoid triplet states, indicating an increased quenching capacity stemming from stronger pigment–pigment interactions [173]. Altogether, these results indicate that subtle modifications within the pigment‐binding pockets, either caused by altering the identity of the Chl ligand or the surrounding and distal residues, can lower the pigment energy levels and create new spectral features. However, it is important to recognize the multifactorial nature of the spectral features of LHCs, which are shaped by the presence of multiple interacting Chl and carotenoid molecules [210]. Furthermore, some Chls are coordinated by water molecules, lipids, or backbone atoms, making them less amenable to modifications via side‐chain substitutions. Instead, certain mutations may destabilize protein folding and/or interfere with pigment incorporation, limiting the accumulation of the holoprotein [174]. Finally, introducing low‐energy states into LHCs could have pleiotropic effects on the overall light‐harvesting process, potentially impacting light‐use efficiency and, thus, plant growth.

To better understand and predict the effects of mutations, researchers have employed molecular dynamics simulations and quantum chemical models to study how factors such as local polarity, conformational flexibility, and the dielectric environment influence pigment site energies and EC [211, 212]. Building on this foundation, machine learning techniques are increasingly being recognized as powerful tools for predictive bioengineering. For instance, networks trained on excitonic energy transfer dynamics can learn complex structure–function relationships and predict how specific mutations may impact pigment coupling and spectral shifts [213]. This approach has the potential to enable high‐throughput *in silico* screening of LHC variants, aiding the identification of promising mutations for experimental validation and accelerating the design of red‐shifted or spectrally broadened antenna complexes.

### Optimizing canopy light‐use efficiency

The photosynthesis‐enhancement resulting from broadened light absorption of leaves is expected to translate into crop applications. However, the direct transfer of technologies from a rosette‐forming plant such as *A. thaliana* to canopy‐forming species is not always straightforward [214, 215, 216]. A more effective approach involves engineering photosynthesis at the canopy level. A “smart canopy” concept should include a vertically stratified and self‐organizing system in which leaves dynamically adjust their photosynthetic capacity based on the local light environment [187]. The spatial heterogeneity in light quality could be addressed by tuning the leaf absorption properties to match the irradiance spectra at various canopy heights or during different plant developmental stages. For instance, this spectral stratification could be achieved via spatiotemporal control of pigment biosynthesis in leaves and/or by selectively expressing red‐shifted LHC isoforms (Fig. 6) [196, 217].

Red‐shifted light‐harvesting systems could be preferentially assembled in the lower foliage, exploiting transcriptional activation by heterologous promoters responding to low R:FR ratio [218], as recently suggested based on simulation of 3D canopy model [198]. Consequently, the identification of FR‐light‐inducible promoters, such as those belonging to phytochrome‐based signaling pathways, is a necessary step toward this goal. These bioengineering strategies might be further supported by modeling approaches that estimate the impact of new leaf phenotypes on overall canopy photosynthetic capacity [217, 219, 220, 221]. Indeed, the predictive power of functional–structural plant models relies on integrating multiple parameters, such as 3D canopy architecture, internal light distribution, ray‐tracing, CO_2_ diffusion rates inside leaf tissues, acclimation of photosynthesis to fluctuating light, and allocation of photosynthates between organs. Modeling could also help quantify the trade‐offs between metabolic investment and yield gains, thus addressing key barriers to develop optimized crop varieties [222, 223, 224, 225, 226, 227, 228, 229].

## Too far (red) to be true?

The absorption of FR light in photosynthesis is a dynamic process spanning several orders of magnitude, from ecosystems to the molecular architecture of photosystems. At present, the term “RFs is used interchangeably to describe a spectroscopic feature (such as a wavelength threshold) or the structural determinant responsible for long‐wavelength absorption. However, the exact localization of RFs cannot be reliably determined solely from structural data, making red‐shifted bands largely volatile spectroscopic attributes [43, 138, 140, 154, 181, 230].

The ability to engineer the spectroscopic features of LHCs is expected to prompt a revision of the current definition of RFs, embracing new types of excitonic interactions deriving from rational design approaches [231, 232, 233, 234]. Nonetheless, efforts to enhance FR light absorption, such as incorporating heterologous Chl types or modifying the LHC protein matrix, must contend with structural and biophysical constraints, which can impact EET dynamics, charge separation, and other feedback regulatory mechanisms [235, 236, 237, 238, 239, 240, 241]. The continuous investigation into the structural and functional properties of LHCs is expected to facilitate the transfer of spectroscopic phenotypes between LHC isoforms. To support this, developing methods to predict excited‐state energy levels of FR pigments and assess the effects of tuning interventions is crucial.

A promising approach involves integrating high‐resolution structural analyses with quantum mechanical calculations to elucidate how subtle protein modifications can affect FR absorption and excited‐state lifetimes, as recently reported for EET pathway analysis in PSII [242]. Finally, the biodiversity of light‐harvesting systems represents an immensely valuable resource for translational research and nature‐based engineering. Phylogenetic comparative methods coupled with structural–functional investigations could help identify and pinpoint factors responsible for long‐wavelength absorption. In particular, the study of organisms adapted to natural, light‐limited environments, such as understory or aquatic habitats, could offer insightful models to guide LHC spectral tuning. This interdisciplinary integration will be essential for developing next‐generation crops capable of dynamic spectral partitioning across canopy layers, ultimately enhancing productivity.

## Author contributions

LD and DM conceived this manuscript. AA, EAC, DM, CB, SC, RC, and ZG conducted the bibliographic review and contributed to the writing. AA, EAC, SC, LD, and ZG prepared the figures. RB supervised the manuscript preparation and edited the final version.
